# Historical Perspectives on Flavivirus Research

**DOI:** 10.3390/v9050097

**Published:** 2017-04-30

**Authors:** Michael R. Holbrook

**Affiliations:** NIAID Integrated Research Facility, 8200 Research Plaza, Ft. Detrick, Frederick, MD 21702, USA; Michael.holbrook@nih.gov; Tel.: +1-301-631-7265

**Keywords:** flavivirus, yellow fever, West Nile, Japanese encephalitis, tick-borne encephalitis, Zika, dengue

## Abstract

The flaviviruses are small single-stranded RNA viruses that are typically transmitted by mosquito or tick vectors. These “arboviruses” are found around the world and account for a significant number of cases of human disease. The flaviviruses cause diseases ranging from mild or sub-clinical infections to lethal hemorrhagic fever or encephalitis. In many cases, survivors of neurologic flavivirus infections suffer long-term debilitating sequelae. Much like the emergence of West Nile virus in the United States in 1999, the recent emergence of Zika virus in the Americas has significantly increased the awareness of mosquito-borne viruses. The diseases caused by several flaviviruses have been recognized for decades, if not centuries. However, there is still a lot that is unknown about the flaviviruses as the recent experience with Zika virus has taught us. The objective of this review is to provide a general overview and some historical perspective on several flaviviruses that cause significant human disease. In addition, available medical countermeasures and significant gaps in our understanding of flavivirus biology are also discussed.

## 1. Introduction

The intent of this review is to provide a broad-brush overview of the flaviviruses, some of their historical highlights and to identify significant gaps in our understanding of these very interesting viruses. The diversity in arthropod-vectors, reservoir species and diseases caused in humans is unlike any other virus family. The recent outbreaks of Zika, yellow fever and Usutu viruses should highlight the potential impact of the flaviviruses to human health while the continuing challenges of dengue, Japanese encephalitis and tick-borne encephalitis reminds us of the number of people at risk for infection with one of these viruses.

## 2. Origins of Flavivirus Research

In the current classification of flaviviruses (Family *Flaviviridae*; genus *Flavivirus;* type species yellow fever virus, Asibi strain) that are associated with infection of mammals, there are two main groupings of viruses: those transmitted by ticks and those transmitted by mosquitoes. The tick-borne flaviviruses are a closely related, monophyletic group consisting of a single “serocomplex”, despite distinct differences in the disease caused by representative viruses. The mosquito-borne viruses are far more diverse, consisting of the Japanese encephalitis virus (JEV) serocomplex, yellow fever virus (YFV) and members of the four dengue virus (DENV) serotypes, among many others. Evaluations of phylogenetic divergence times indicate the origins of the flaviviruses go back around 100,000 years to a common ancestor with the split between mosquito- and tick-borne flaviviruses around 40,000 years ago [1]. The flaviviruses were originally grouped among the togaviruses based on early serological assessment, but were separated from the togaviruses into the family *Flaviviridae* in 1984 based on differences in structure, gene sequence and replication strategy [2]. Since that time, delineation of the viral genome, virus structure and viral biology have identified significant differences between the flaviviruses and their historical colleagues in the family *Togaviridae*. In addition, several viruses have been identified as “non-vectored” flaviviruses and a number of insect-specific flaviviruses (ISFs) have also been discovered [3]. Dual-host affiliated ISFs (dISFs) were first identified in 2004 and, to date, have only been identified in mosquitoes [3]. While the ISFs are genetically distinct from other flaviviruses, the dISFs cluster with the vertebrate associated flaviviruses suggesting a certain level of evolutionary congruence.

**Figure 1:**
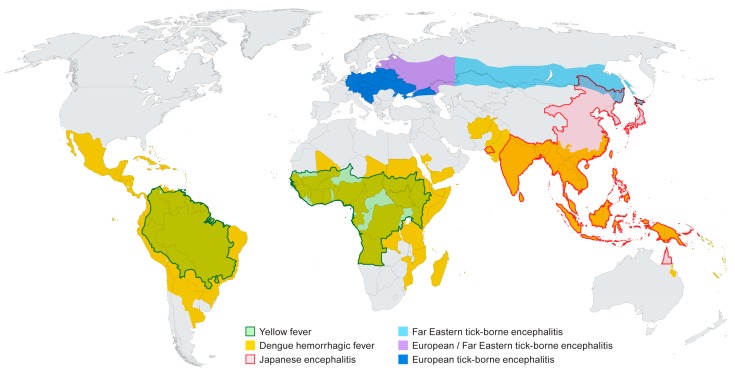
Distribution of major flaviviruses discussed in this article. Information was adapted from data and figures provided on Centers for Disease Control and Prevention (CDC) and World Health Organization (WHO) websites.

A number of flaviviruses are considered major human pathogens (Figure 1) and the diseases they cause have been recognized for many years. The mosquito-borne viruses can broadly be grouped as those transmitted by *Culex* spp. mosquitoes (JE serocomplex) and generally associated with neurotropic viruses, and those transmitted by *Aedes* spp. mosquitoes and more closely associated with viscerotropic or hemorrhagic disease in humans. The recent discovery that Zika virus (ZIKV), a virus transmitted by *Aedes aegypti*, can cause severe neurological disease in developing fetuses may stimulate a re-evaluation of the impact of infection by the closely related DENV or YFV on pregnant women. The tick-borne flaviviruses cause of number of significant diseases in humans that are typically associated with neurological symptoms although hemorrhagic manifestations have been documented following infection with some of these viruses. The diversity of arthropod vectors, disease characteristics and the wide geographic distribution of the flaviviruses makes these viruses especially interesting, particularly if one considers that most people throughout the world live in a flavivirus endemic region. The relative ease with which some of these viruses can be introduced into new environments should also raise concerns and highlight the need for extensive additional research on these viruses, both in the lab and in the field.

### 2.1. Yellow Fever

As far back as the mid-1600s the “Black Death” or “Blood Vomit” (Xekik in Mayan) was a known disease that afflicted people primarily in port cities throughout the Caribbean and in the Americas. Outbreaks of this disease were documented in cities in Europe, particularly along the Mediterranean coast where the disease was thought to be imported from Africa. Although evidence of a yellow fever (YF)-like disease was reported in Hispaniola in 1495 [4], the first documented outbreak of yellow fever was either in Barbados [5] or St. Christophe (now St. Kitts) in 1647 [4]. The outbreak in the Caribbean subsequently spread to the Yucatan peninsula in 1648 [4]. After 1648, YF spread throughout the Caribbean, including regular outbreaks in Cuba. In 1793, a significant outbreak of yellow fever hit Philadelphia and killed around 10% of the population [6]. Perhaps the largest outbreak of yellow fever in the Americas hit communities along the southern Mississippi river in 1878 between Memphis and New Orleans. This outbreak killed upwards of 20,000 people with estimates of around 120,000 cases [7]. Outbreaks of yellow fever persisted in the United States through 1905 when the final outbreak was document in New Orleans. A number of clinical descriptions of yellow fever disease have been published, including the extensive description of the Philadelphia outbreak by Benjamin Rush [6]. The cause of yellow fever was unknown and was frequently referred to as a “miasma” transmitted by foul air [7]. A very detailed account of the historical epidemiology of YF was provided by Henry Rose Carter and is available online [4].

In 1897 the Yellow Fever Commission was established to investigate the origins of YF in Cuba. Previous work by J.C. Nott and Carlos Finlay had suggested that mosquitoes may be a means of transmitting the disease YF between people [8,9]. The Yellow Fever Commission established that mosquitoes were, in fact, the vector for the disease [10]. Members of the Commission and other military “volunteers” participated in a series human infection studies to demonstrate that mosquitoes moved the disease from afflicted patients to healthy study participants, in some cases, at the cost of their lives. These studies were pivotal in proving the role of mosquitoes in the transmission of YF and validated the hypotheses of Nott and Finlay from many years earlier.

The concept of “viruses” was not understood at the time of the Yellow Fever Commission. Instead, the prevailing hypothesis was that a bacterium (*Bacillus icteroides* or *Leptospira icteroides*) was the transmissible element causing YF as some studies identified bacteria in cultures taken from YF patients. It was not until 1928 when Stokes and colleagues, in a seminal series of studies, identified a “filterable” agent as the transmissible component of YF infection that the concept of a “yellow fever virus” was understood [11].

Infection with YFV can result in disease ranging from a sub-clinical infection to severe hemorrhagic disease and death [12]. In the more severe forms of the disease, the illness is typically biphasic, progressing from an “infection” phase, through “remission” and into a period of “intoxication”. The “infection” phase of disease presents as a “flu-like” illness with fever, malaise, headache and myalgia, but is complicated by hyperemia, conjunctival injection and tenderness in the liver. Many patients recover following the “infection” phase, but others progress though a brief period of remission where symptoms subside, and into the very severe period of “intoxication”. This phase of the disease is characterized by hemorrhagic disease and multi-organ dysfunction with symptoms including the characteristic jaundice, nausea, vomiting and frank hemorrhagic manifestations. Terminal patients can develop neurological manifestations, including delirium, convulsions and coma. Neurological symptoms are likely due to generalized inflammatory responses and vascular leakage into the brain rather than a specific neurotropic characteristic of the virus.

The specific mechanisms of YFV induced disease are unclear. Liver dysfunction is evidenced by jaundice and significant changes in liver enzyme profiles and hemorrhagic indications are frequently apparent in extremely ill patients [13]. Unlike some viruses that cause hepatitis by stimulating an inflammatory response, YFV directly infects hepatocytes and Kupffer cells [14,15,16] leading to a loss of hepatocyte function and acute liver injury. YFV infection can also significantly impact the vascular endothelial cell barrier, but it is not clear whether the onset of vascular leakage is due to changes in liver physiology, inflammatory cytokine response, direct infection by YFV or by an another mechanism [13]. The loss of liver function may also lead to dysregulation of the coagulation cascade, but specific details have not been determined. There have been reports of disseminated intravascular coagulation (DIC) in YF patients and global loss of coagulation factors in YFV infected rhesus macaques has also been reported [17,18]. Much of what is known about YF pathogenesis is the result of a handful of clinical assessments, a few studies in the macaque model and extrapolation from clinical cases of dengue hemorrhagic fever, which is caused by a related virus, but is not the same disease. There are clearly a number of significant questions that need to be addressed to gain a better understanding of YFV pathogenesis.

### 2.2. Dengue

“Dandy Fever” and break-bone fever were described as early as the late 1700s and pandemics of what was called “dengue” were seen approximately every 50 years from the 1770s through 2005, as described in an intriguing article by Scott Halstead [19]. Halstead suggests that many of the early descriptions of “dengue” were instances of Chikungunya virus (CHIKV) infection as the disease was frequently described as having an “arthritic” component of the disease that persisted once fever had waned. Dengue, caused by DENV infection, was recognized as a disease separate from the “dengue” caused by CHIKV infection with the significant differences being DENV infection was referred to as “break-bone fever” and the presence of headaches, a rash and without the arthritic sequelae [19]. The “official” first description of dengue, or “joint fever” was by David Bylon following an outbreak in Java in 1779 [20]. In the United States, an extensive outbreak occurred in the southern part of the country in 1922 where it was estimated that 1–2 million people were impacted by this disease [21,22].

The hypothesis that YF and dengue (diseases) were transmitted in the same manner was recognized in the 1800s; Dr. William Smart noted “…there were those who attributed its (dengue) diffusion to a widely spread so-called ‘epidemic constitution of the atmosphere’, such as was at the same period maintained to be the sole cause of epidemic yellow fever” [23]. It was also noted that dengue and yellow fever occurred in the same locations and that those having the “milder fever” were not immune against developing yellow fever. Shortly after the discovery that YF was transmitted by *A. aegypti*, dengue was also shown to be transmitted by this mosquito vector [24,25,26,27] and that the transmissible agent was a “filterable agent” [28]. In what has complicated the management of dengue in years since, it was also discovered that *A. albopictus* is a vector for transmission of dengue [29].

Using “cross-immunity” and “dermal neutralization” tests in addition to “intracerebral neutralization” tests with sera from convalescent human volunteers, Sabin and Schlesinger demonstrated that there were at least two different “immunological types” of dengue virus [30]. Subsequent serological assessments, including hemagglutination and complement fixation assays, were used to further distinguish DENV serotypes and to demonstrate that the DENV were composed of four distinct virus serotypes [31,32,33,34]. While serologically distinct, viruses from each serocomplex cause similar disease in humans. Subsequent genetic analysis, initially by oligonucleotide fingerprint analysis [35] and later by partial and full genome analysis [36,37,38] have validated serological assessments by identifying four distinct virus genotypes that correlate with virus serotypes and that are divergent by no more than 6% at the nucleotide level [39].

People at risk for dengue disease inhabit tropical and subtropical regions around the world with an estimated 40% of the global population at risk for DENV infection and 390 million cases annually [40]. Dengue is a disease with a range of clinical presentations. In an effort to harmonize the clinical description of dengue by clinicians, in 2009 the WHO developed a classification system for dengue that graded the severity of disease based on clinical observations [41]. DENV infection is predominantly seen as an acute febrile disease that can last up to a week from onset of symptoms and may also follow a biphasic course. This disease, termed dengue fever (DF), is also characterized by headache, myalgia, lumbosacral pain and arthralgia of variable severity. Characteristic in many cases is a macular rash that appears early in the infection that may progress to a secondary rash. Hemorrhagic manifestations including petechiae and other hemorrhagic signs may occur, but are less common [27,42]. Severe dengue takes the form of dengue hemorrhagic fever (DHF) which has four severity grades, with the more severe grades (III and IV) classified as dengue shock syndrome (DSS) [43]. In DHF, patients typically have hemorrhagic manifestations, including petechial hemorrhage, whereas those that progress to DSS have evidence of mild shock with failure of the circulatory system (Grade III) or profound shock with no pulse or blood pressure (Grade IV). The development of DHF/DSS correlates with the onset of thrombocytopenia, prolonged clotting times and other characteristics of DIC. While the occurrence of DHF/DSS can occur in any DENV infection, it appears to be more frequent in secondary DENV infections, particularly in children or in newborns who are partially protected by maternal antibodies [44]. In practical application, the grading system of severe DENV infection is not as clearly defined as the classification scales imply. Subsequently, efforts are being made to improve the classification on severe dengue [45].

There have been a number of studies evaluating the role of antibody dependent enhancement (ADE) and its role in development of severe dengue disease. The prevailing hypothesis is that the presence of low-affinity, cross-reactive, non-neutralizing antibodies from a primary DENV infection (i.e., with one serotype) will enhance DENV infection with a second serotype (heterotypic). The premise is that once the non-neutralizing antibodies bind virus, they then bind a cell presenting an Fc receptor to facilitate virus entry into that cell. The fact that the virus is not “neutralized” in the antibody-virion complex allows the virus to release viral RNA and leads to a productive infection. The occurrence of ADE may be exacerbated by the occurrence of “original antigenic sin” wherein the response of T cells to secondary DENV infection may increase the potential for severe disease [46]. For more complete discussions on the role of ADE in DENV infections, please see reviews by Halstead [47] and Flipse et al. [48]. This topic is particularly pertinent at the current time as there are discussions regarding the possibility that antibodies specific for DENV may be cross-reactive for related viruses (e.g., ZIKV) leading to enhanced disease [49,50].

### 2.3. Japanese Encephalitis

Epidemics of encephalitis had been noted in Japan as far back as 1871. In 1924, an outbreak of encephalitis that affected 6000 people and killed 60% of those affected, gave rise to a disease called Japanese B summer encephalitis [51]. The agent causing Japanese B summer encephalitis, subsequently termed Japanese encephalitis virus (JEV), was isolated and characterized in non-human primates in 1933 [51,52] and a number of additional isolates were made in mice during an outbreak in 1935 [53]. Japanese B encephalitis was classified among the “B” type togaviruses based on serological studies. Genetic analysis of JEV genomes suggests that the virus originated in the Malay Archipelago several thousand years ago and then spread throughout Asia [54]. There are four distinct genotypes of JEV that have been circulating throughout Asia for the past 50 years. Recently, several isolates have been made of viruses representing the 5th genotype of JEV, which had previously been represented by a single isolate from 1952 [55,56,57].

There are an estimated three billion people who live within areas of 24 countries impacted by JEV [58]. The annual incidence of JE is around 70,000 cases with a case fatality rate estimated to be 14,000–20,500 per year [59,60]. In countries where JEV is endemic, the incidence rate is 0.6–12.6/100,000 depending upon geographic and climatic factors in addition to vaccination rates in susceptible populations [59,60].

JEV is transmitted by *Culex* spp. mosquitoes in an enzootic cycle that includes pigs and birds [52,61]. Pigs are an important component of the transmission cycle as an amplifying host as they can develop a high titer and long-lasting viremia that does not seem to have a significant health impact on these animals [61,62]. The involvement of birds in transmission of JEV is less relevant to direct transmission to humans than it is to the dissemination of the virus to new geographic areas.

Infection with JEV causes an acute non-specific febrile illness that consists of rapid onset with headache, myalgia, diarrhea and vomiting. In some patients, the disease can be complicated by neurological signs including opisthotonus, acute flaccid paralysis, convulsions, mental confusion, mask-like facies and cogwheel rigidity [63]. Severe disease can progress to severe encephalitis, meningitis, loss of conscious, coma and death. Neurological sequelae occur in about 30% of those who survive severe disease. These sequelae can include seizures, physical disabilities and cognitive deficits [59,64]. An extensive description of the clinical features of JE can be found in a book chapter by Scott Halstead [51].

### 2.4. Tick-Borne Encephalitis

Tick-borne encephalitis (TBE) was first recognized in 1932 as a severe neurological disease that occurred in forest workers in the far eastern Soviet Union (now Russia). In 1936 the Soviet Union established an exploratory expedition to determine the source and cause of this disease. As a result of this expedition, *Ixodes persulcatus* was identified as the vector for TBE [65]. In 1937, individual groups identified the causative agent of TBE to be a virus that was subsequently called “far-eastern encephalitis virus” [65]. A similar, but less severe, disease that was found in western Russia and Eastern Europe was called Western encephalitis [66]. Western encephalitis was also known as “biphasic milk fever” given its apparent linkage to consumption of unpasteurized milk from infected animals. The causative agent for Western encephalitis was identified during outbreaks in Czechoslovakia as a virus related to the far-eastern TBE virus (TBEV) [67]. Western encephalitis virus (subsequently known as central European encephalitis virus) is transmitted by the *Ix. ricinus* tick. Over the course of outbreak investigations, a third variant of TBEV was identified and termed the Siberian subtype of TBEV. This virus, transmitted by *Ix. persulcatus*, caused a disease that was of intermediate severity between far-eastern TBEV and its European relative. Genetic analysis of far-eastern TBEV, Siberian and central European encephalitis virus demonstrated that these viruses were closely related not only serologically and in the clinical disease they caused, but also genetically [68,69]. The three viruses are now called TBEV-FE (Far-Eastern), TBEV-Sib (Siberian) and TBEV-Eu (European) [70].

The TBEV are maintained in a life cycle that includes their tick hosts and small rodents upon which the ticks feed. While TBEV can be maintained in tick-populations by trans-stadial and trans-ovarial transmission [71], horizontal transmission via co-feeding of ticks on small mammals may also play a significant role in maintaining the virus in ticks [72]. In support of co-feeding transmission, recent studies with Powassan virus (POWV) have shown that a potential mammalian host for POWV, *Peromyscus leucopus*, did not develop disease when infected with POWV and did not develop a sufficient viremia to directly infect feeding ticks [73]. 

Infection with TBEV-FE can cause a very severe disease following an uneventful prodrome. The disease manifests with a rapid onset, high fever, myalgia and neurological indications including headache, photophobia and clinical evidence of encephalitis or meningitis with complications including flaccid motor neuron paralysis, ascending paralysis or hemiparesis [74,75]. The case fatality rate for TBEV-FE infections is 20–30% with many survivors having long-term neurological sequelae including paresis and atrophy of the neck and brachial plexus muscles, paresis within the lower extremities as well as poliomyelitis-like neurological sequelae [74].

Unlike TBEV-FE infections, the disease caused by TBEV-Eu can be relatively mild with a number of infections resulting in subclinical infections [76]. In those who develop clinical disease, it is typically biphasic with the first phase represented as a “flu-like” illness with fever, myalgia and malaise. In about 65% of symptomatic cases, the clinical course resolves after the first phase. For those who progress to the second phase, high fever and neurological involvement including meningitis and meningoencephalitis are typical symptoms [77]. The case fatality rate for TBEV-Eu infections is 1–2% with long-term sequelae atypical except for in older patients [75].

Infection by TBEV-Sib results in a disease that is described as intermediate between those caused by TBEV-FE and TBEV-Eu. However, a unique characteristic of this virus is that it has been associated with chronic infection in both humans and non-human primates, a complication not typically described for TBEV-FE or TBEV-Eu [76,78,79,80,81,82].

Since the initial discovery of TBEV, a number of related viruses causing human disease have been identified including Omsk hemorrhagic fever virus (OHFV), Kyasanur forest disease virus (KFDV), Alkhumra hemorrhagic fever virus (AHFV) and POWV. OHFV is found in a small region near Novosibirsk in Russia [83], KFDV is found in an ever-expanding range in India [84] and its closely related cousin (AHFV) is found primarily in Saudi Arabia along the coast of the Red Sea [85]. POWV has been suggested as the most ancestral member of the TBEV serocomplex [1,69] and is the only tick-borne flavivirus found in the Americas. Gritsun et al. provide a comprehensive review of TBE [76].

## 3. Emergence/Re-Emergence

In August 1999, the introduction of West Nile virus (WNV) into the USA [86] significantly heightened awareness of vector-borne viruses. While vector-borne viruses are endemic and common in many parts of the world, the introduction and rapid spread of WNV across the USA [87] was an awakening for many medical and government officials. The expansion of WNV distribution was not limited to the United States. West Nile virus has also spread into Central and South America, parts of Europe and Russia [88]. Since 1999, WNV has become globally distributed and causes severe disease and death worldwide. Recent reviews by Chancey et al. and Kilpatrick discussed the spread of this virus and its impact on the health of humans and wildlife [89,90].

Concurrent with the spread of WNV in the United States was the recognition of an increase in cases of POWV or Deer Tick virus (DTV) infection [91], perhaps due to increased surveillance and testing of clinical cases of encephalitis. In 2005 and 2013, cases of DENV infection were identified in the Brownsville, TX, along the southern border with Mexico [92,93], in 2009–2011, several cases of dengue were identified in south Florida [94] and in 2001 DENV was seen in Hawaii for the first time in nearly 70 years with over 120 cases identified during the outbreak [95]. A subsequent outbreak of dengue fever was reported in Hawaii in the fall of 2015 with over 100 cases confirmed [96]. It has also been reported that DENV was circulating in the area of Houston, TX in 2003–2005 [97]. The Houston metro area is home to over 6.5 million people and potential vectors for DENV are abundant. 

In 2001, Usutu virus (USUV) was identified in Austria [98], the first time this virus has been found outside of the African continent. Usutu virus has subsequently spread to a number of different countries throughout Europe and serological testing suggests its presence in other countries [88,99,100]. While the presence of USUV has primarily been detected through ecological surveillance in mosquito and bird populations, symptomatic human cases of USUV infection have been found in Italy and Croatia [101]. The frequency of symptomatic infection of humans by USUV appears to be very low with only a handful of known cases reported since the discovery of this virus in 1959 [102].

The expansion of ZIKV into the Americas has again highlighted the importance of surveillance and of disease reporting and recognition. For many months, clinicians in Brazil were reporting increases in the occurrence of microcephaly in newborns and a potential correlation with ZIKV infection. These reports were met with a healthy dose of skepticism. Now we have come to realize that there is a correlation between microcephaly and ZIKV infection and that the impact of ZIKV infection is much different from other flaviviruses. A more extensive review of ZIKV is presented elsewhere in this Special Issue.

## 4. Vaccines

There are a number of highly efficacious vaccines for protection against flavivirus infection, including, perhaps, the best vaccine ever produced. In 1937, Max Theiler reported production of the YFV 17D vaccine [103]. This vaccine was generated by serially passing the type strain Asibi in mouse and chicken tissue to produce the attenuated and non-neurovirulent vaccine virus [104]. Since its introduction, over 600 million doses of the 17D vaccine have been delivered [104] with no documented evidence of vaccine reversion to wild-type virus. A single vaccination with the 17D virus provides rapid protection (people are considered immune 10 days post-vaccination) and potentially life-long protection [105]. Serological protection against YFV infection has been defined as having a log_10_ neutralization index of >0.7 (or a dilution titer of 1:10) [106]. While this threshold has not been empirically proven effective in the case of all flaviviruses, it is generally accepted as the minimum requirement to demonstrate efficacy for all flavivirus vaccines. The 17D vaccine played a critical role in limiting the scale of a 2016 YF outbreak in Angola and the Democratic Republic of Congo where over 18 million doses were given to stop the outbreak. A significant challenge with the 17D vaccine, however, is that it is grown in Specific Pathogen Free eggs, which limits rapid expansion of virus production. A number of cases of vaccine-related viscerotropic or neurotropic disease have been reported following vaccination with YFV 17D [107,108], but these cases appear likely due to co-morbidities impacting immune competence [107]. While the specific cause(s) of vaccine-related disease are unknown, their occurrence has inspired the development of potential vaccine candidates to replace the 17D vaccine. These include adenovirus-based [109], vaccinia-based [110] (ClinicalTrials.gov NCT02743455) DNA-based [111,112], and inactivated vaccines [113] (ClinicalTrials.gov NCT00995865).

The first JE vaccine produced and introduced in Japan in 1954 was a formalin-inactivated vaccine using mouse brain homogenates from JEV-Nakayama infected mice [114,115]. Since the initial introduction, several modifications have been made to the JEV vaccine including efforts to remove brain material, increasing purity and shifting the strain from Nakayama to Beijing-1 in some countries [114]. The mouse-brain derived vaccine was discontinued in 2011. At the current time, there are a number of JE vaccines in use. Use restrictions vary somewhat depending upon the vaccine, but in general, all appear to be effective in those vaccinated who are over one year old [64]. Two vaccines are based on the attenuated SA14-14-2 JEV, an inactivated cell culture vaccine broadly marketed as Ixiaro^®^ or IC51 (Valneva, Vienna, Austria), and a vaccine composed of the live SA-14-14-2 virus itself that is available in China and other Asian countries [64]. An inactivated vaccine based on the Beijing-1 virus is available in Japan under the trade names JEBIK V (BIKEN, Kagawa, Japan) or ENCEVAC (Chemo-Sero Therapeutic Research, Kumamoto, Japan) [64]. A recently developed chimeric vaccine based on the YFV 17D virus back-bone and containing the prM and E protein genes from JEV SA14-14-2 (ChimeriVax-JE) (Sanofi-Pasteur, Lyon, France) is available in Thailand and Australia [64].

The first vaccine for TBEV was an inactivated virus vaccine that was produced shortly after the virus was discovered in 1937 and was used to vaccinate workers during the course of outbreaks [67]. Vaccines using cell culture systems were developed in the 1960s [116] and early 1970s. Early vaccines generated in the Soviet Union were based on a Far-eastern subtype of TBEV. The first vaccines generated in Europe were based on the European strain Neudörfl grown in primary chicken embryo cells [117] and marketed as FSME-IMMUN^®^ (Baxter AG, Vienna, Austria) In the 1980s, a similar European vaccine was produced using the K23 strain of TBEV and is marketed as ENCEPUR^®^ (Novartis/GlaxoSmithKline, Germany) [118]. Both European vaccines have undergone modifications over the years to improve safety and immunogenicity. Currently both the FSME-IMMUN^®^ and ENCEPUR^®^ have similar formulations and both are highly immunogenic and clinically effective [119,120]. Neither FSME-IMMUN^®^, nor ENCEPUR^®^ are licensed for use outside of Europe despite extensive demonstration of safety and clinical efficacy. In addition to the European vaccines, Russian scientists have generated two vaccines for use against TBEV by utilizing either TBEV-FE strain Sofjin or strain 205 [119].

The development of a vaccine for DENV has been an ongoing effort for several decades. Vaccine development is complicated by the need for the vaccine to be protective against all four DENV serotypes. The potential for enhanced disease during secondary DENV infections could be exacerbated if a vaccine is not fully protective against all four serotypes. At the current time, there are several vaccines that show promise, including CYD-TDV (or Dengvaxia^®^) from Sanofi-Pasteur (Lyon, France). The CYD-TDV has been through two phase 3 clinical trials and has been approved for use in individuals aged 9–45 years living in endemic areas of Mexico, Brazil, the Philippines, El Salvador and Costa Rica [121,122]. The CYD-TDV vaccine contains four live chimeric viruses that consist of the YFV 17D virus backbone, but has swapped in the membrane and envelope protein genes for the individual DENV serotypes in place of those for YFV [123]. Phase 2 and phase 3 clinical trials with this vaccine have shown this vaccine to induce robust immune responses in children and adults against all four serotypes when following a three-dose vaccination schedule [124,125]. Clinical trials were carried out in DENV endemic countries. There are a number of caveats regarding participant serostatus against flaviviruses at the start of the trials and participant age that may have impacted the study [125]. Analysis of data from trials compiled through early 2016, led the WHO Strategic Advisory Group of Experts on Immunization (SAGE) to provide recommendations for the administration of the CYD-TDV vaccine [126]. These recommendations stipulate that the vaccine should only be given in populations where DENV seroprevalence is >70%, although it may be effective in populations with 50–70% seroprevalence. The vaccine should be given in a three-dose vaccination schedule and it is not recommended for children <9 years old due to an increased risk of hospitalization and severe disease. The risk to young children in endemic areas is due to the presence of existing cross-reactive antibodies of maternal origin in neonates, or virus exposure in older children. The fact that the CYD-TDV vaccine cannot be given to young children to provide complete protection against DENV infection is frustrating, but highlights an additional challenge of producing an effective vaccine for dengue. While this vaccine appears to have some challenges [127], analysis of clinical trial data suggests that routine vaccination programs utilizing the CYD-TDV could have a significant impact on the number of dengue cases in endemic areas [121]. Despite its limitations, the CYD-TDV vaccine does represent a significant step forward in the effort to reduce the impact of DENV infection.

In addition to the CYD-TDV vaccine, several other DENV vaccines are in clinical trials. A tetravalent vaccine (DENVax) developed by Takeda Vaccines (Singapore) is based on work initiated at CDC-Fort Collins a number of years ago. DENVax is similar to the CYD-TDV vaccine, except that it uses an attenuated DENV-2 virus backbone rather than YFV to generate chimeras for DENV-1, -3 and -4 and uses the authentic attenuated virus for DENV-2 [128,129]. The DENVax vaccine has been shown to be safe and immunogenic in phase 1 clinical trials [130] and is currently being evaluated in phase 2 and 3 clinical trials [131].

The TV003 vaccine (NIAID/Johns Hopkins) (Maryland, USA) is an admixture of four recombinant DENV that have mutations in the 3′-non-coding region leading to attenuation of the viruses [132]. Early studies demonstrated development of protective immunity following vaccination [133]. In a human DENV challenge trial, TV003 was shown to be protective when volunteers were challenge with an attenuated DENV-2 virus six months after vaccination [134]. One side effect of the TV003 was the development of a mild rash. More recent trials with a different admixture, TV005, have shown very promising results [135]. This formulation also seems to have resolved the issue of the post-vaccination rash seen in TV003 trials. A phase 2 trial for the TV003 vaccine is currently underway (ClinicalTrials.gov NCT02332733) while phase 1 and efficacy trials for a different formulation of this vaccine are in development (NCT02879266; NCT02873260). 

In addition to the above-mentioned vaccines, there are a number of inactivated, subunit or DNA vaccines currently in development or in clinical trials. For more comprehensive reviews on DENV vaccines and the issues related to vaccine development, please see articles by Thomas and Rothman [136], Thomas [137] and McArthur et al. [124].

**Figure 2:**
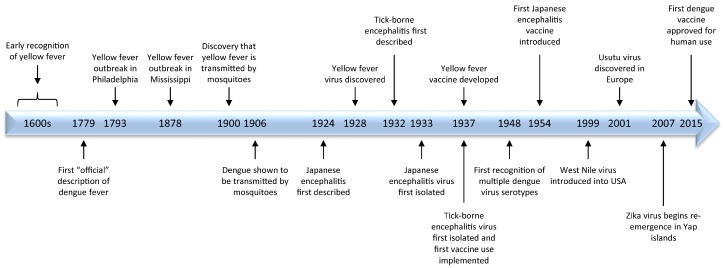
A time-line of historical highlights of flavivirus research.

Major milestones in flavivirus research are highlighted in Figure 2.

## 5. Gaps in Flavivirus Research

There are a number of significant gaps in our understanding of flaviviruses and the diseases they cause. Comparatively, more is known about DENV due to its broad distribution, significant health impact and the lower biocontainment level (BSL-2) required for safely performing research with this virus. The recent global spread of ZIKV has provided impetus and financial support for research with this interesting virus which has allowed for significant advances in our understanding of ZIKV structure, pathogenesis and for vaccine development in a short period of time. However, many questions remain regarding fundamental components of flavivirus biology.

### 5.1. Identification of the Cognate Receptors for Flaviviruses

Given the divergent diseases caused by the different flaviviruses, it is probable that there is not a single receptor for all of the viruses, but rather a family or groups of structurally similar cell surface proteins that function as receptors for these viruses. Receptor proteins probably have different functions and cell expression patterns, given that there appears to be variable target cell types between different flaviviruses. For example, most of the mosquito-borne flaviviruses will infect both Vero and C6/36 cells, while tick-borne flaviviruses do not easily infect C6/36 cells and many replicate poorly in Vero cells. Previous work has suggested that highly glycosylated molecules, such as DC-SIGN (Dendritic Cell-Specific Intercellular adhesion molecule-3-Grabbing Non-integrin), may be receptors for flaviviruses. However, interactions with molecules such as DC-SIGN appear to be low affinity interactions rather than high affinity and are not the sole component required for viral entry [138,139]. Furthermore, published work suggests that the flaviviruses typically enter cells via receptor-mediated endocytosis, suggesting a specific receptor-ligand interaction. Interestingly, a recent study with YFV demonstrated that the 17D vaccine strain and wild-type Asibi virus utilized different mechanisms for cell entry, potentially suggesting alternative receptors for two nearly identical viruses [140]. In addition, an important point to consider is that the receptor to which a virus binds may be a critical component of the response to viral infection as many cell surface proteins have signaling functions that could impact permissiveness to viral entry replication. 

### 5.2. An Understanding of the Role of T Cell in Mediated Immunity in Flavivirus Infection

With the exception of dengue, research efforts toward understanding of the role of T cell mediated immunity during flavivirus infection in humans has only recently become of significant interest. In addition to extensive work with dengue virus, a number of studies with WNV and TBEV have evaluated T cell responses to wild-type virus infection. The majority of work with WNV has focused primarily in mouse models [141,142]. While these studies provide considerable detail regarding mechanisms of the host response in mice, they are of limited utility as it is not clear how findings in these mice apply to the human condition. Work focused on stimulation of T cell immunity following TBEV infection has been very limited [143,144,145]. Studies with YFV have evaluated the importance of T cells in response to vaccination [146,147,148]. The broad distribution and number of DENV infections has allowed evaluation of T cell mediated immunity in patients with dengue, dengue hemorrhagic fever or dengue shock syndrome since the early 1990s. These studies have found that the role of T cell immunity during DENV infections is complicated. Some studies have suggested a limited role for T cells in primary DENV infection while others suggest that the T cell response correlates with the severity of disease [46]. As mentioned previously, the role of T cells during secondary DENV infection may contribute to enhanced disease through expansion of low avidity cross-reactive T cells. For a more thorough review of the role of T cells in DENV infection, see a review by Screaton et al. [46].

### 5.3. Animal Models that Faithfully Recapitulate Human Disease

The animal models routinely used for evaluation of flavivirus pathogenesis and the testing of medical countermeasures are not generally representative of disease as it manifests in humans. Mouse models for neurotropic flaviviruses develop neurological disease, but the disease is typically monophasic and lethal, depending upon virus inoculum and mouse strain, within 7–14 days. Primate models for neurotropic flaviviruses are also limited as most do not develop disease when the virus is delivered peripherally [149]. In the case of TBEV, non-human primates (NHP) are susceptible to infection, develop a disease similar to a mild case of human infection and have been shown to develop chronic infection [150].

Our understanding of YFV-induced pathogenesis is limited by a lack of useful animal models. As established by Stokes et al. [11], the rhesus macaque develops disease similar to that seen in humans, but the disease course is monophasic and of shorter duration [18]. Experimental work with non-human primates is also limited due to the cost associated with working with these animals. A hamster model for YFV was identified in the early 2000s by adapting either the Jimenez or Asibi strains of YFV by serial passage in hamsters [151,152]. Animals infected with the adapted viruses develop disease similar to that seen in humans, but this model is limited by the availability of reagents that would help to understand the immunopathology of the disease. More recently, the AG129 mouse, an interferon-deficient strain, was found to develop disease similar to humans when infected with a non-adapted virus [153,154]. Given the limited innate immune response engendered by the lack of interferon, the applicability of the AG129 mouse to understanding human disease is up for debate.

Similar to the case for YFV, there are a limited number of animal models for DENV that are useful for studying human disease. The rhesus macaque may be a faithful model for mild cases of dengue in that they have a productive infection with reasonably high viremia, but do not have the clinical picture that is typically seen in humans. The laboratory mouse is also a poor model for DENV pathogenesis. Since the early 2000s, several modified mouse models have been developed to address specific questions of DENV pathogenesis. These include animals that have some component of their innate immune response, specifically interferon-related, removed or are humanized mouse models. While not necessarily accurately representing human disease, each of these models provides information regarding aspects of DENV-induced pathophysiology that might be applicable to humans. For comprehensive reviews of the existing models for DENV infection see Sarathy et al. [155] and Chan et al. [156].

### 5.4. The Role of Sexual Transmission in Flavivirus Infection

The discovery that ZIKV can be transmitted sexually and that virus appears to persist in semen for some time [157,158] was novel, as flaviviruses had not previously been shown to be transmitted sexually. However, the question to be asked is whether anyone looked. Presumably, if sexual transmission had played a significant role in flavivirus dissemination, it would have been noticed. The discovery of sexual transmission in the case of ZIKV, suggests the ability of other flaviviruses to persist in immune-privileged sites and to be transmitted sexually. This phenomenon clearly needs to be investigated further.

### 5.5. Mother to Child Transmission

The discovery that ZIKV could be transmitted from mother to fetus and cause neurological disease in the fetus was an alarming discovery. The mechanism the virus uses to cross the placental barrier is still being evaluated, but this virus infects a number of cells in the placenta [159]. Previous studies with WNV have suggested that this virus might also be able to cross the placental barrier [160,161], demonstrating that further evaluation of this mechanism of transmission is warranted with all of the flaviviruses. Viable ZIKV has also been identified in breast milk and may be a potential source of transmission from mother to child [162]. The transmission of other flaviviruses from mother to child via breast-feeding has been suspected or documented for several other flaviviruses [163,164,165].

### 5.6. No Therapeutic Options

To date, there are no therapeutic options for the treatment of any flavivirus infection. Supportive care is the norm and has shown some success for the treatment of flavivirus infection, particularly yellow fever and DHF/DSS where fluid loss is a critical concern due to vascular leakage. The lack of treatment options is a significant problem as is evidenced by the continuing dilemma with DENV infections, the recent outbreaks of YFV in Angola and Brazil, and the continuing spread of ZIKV. The typical approach to drug screening focuses on direct antiviral effects in cultured or primary cells. In the case of most flavivirus infections, once symptoms are apparent the virus has been cleared from the blood (no viremia), is seeded in tissues and the host immune response is a significant contributor to the disease. In order to develop successful therapeutic approaches for treatment of flavivirus infections, a combination approach utilizing antivirals and host response directed countermeasures might be required.

## 6. Summary

The objective of this review was to provide a broad overview of flaviviruses and the diseases they cause. A secondary objective was to heighten awareness of the risks of flavivirus induced disease, the potential for continued spread of these viruses and the relative lack of understanding regarding the mechanisms these viruses use to cause disease. Diseases caused by the flaviviruses have been recognized for more than 200 years, but there is a lot of work yet to do before we have a proper understanding of these very interesting viruses.
